# The mitochondrial genome of pin-tailed snipe *Gallinago stenura*, and its implications for the phylogeny of Charadriiformes

**DOI:** 10.1371/journal.pone.0175244

**Published:** 2017-04-06

**Authors:** Chaochao Hu, Chenling Zhang, Lei Sun, Yi Zhang, Wenli Xie, Baowei Zhang, Qing Chang

**Affiliations:** 1 Analytical and Testing Center, Nanjing Normal University, Nanjing, Jiangsu, People's Republic of China; 2 Faculty of Life Science and Chemical Engineering, Jiangsu Second Normal University, Nanjing, Jiangsu, People's Republic of China; 3 Department of Biological Engineering, Utah State University, 4105 Old Main Hill, Logan, UT, United States of America; 4 Jiangsu Key Laboratory for Biodiversity and Biotechnology, College of Life Sciences, Nanjing Normal University, Nanjing, Jiangsu, People's Republic of China; 5 Anhui Key Laboratory of Eco-engineering and Bio-technique, School of Life Sciences, Anhui University, Hefei, Anhui, People's Republic of China; Sichuan University, CHINA

## Abstract

The Charadriiformes, among the most diverse orders of bird, is a good source to research on evolution. The mitochondrial genome sequencing database has rapidly increased in recent years, while Charadriiformes mitogenome has not been well studied. In this research, we determined the complete mitogenome sequence of *Gallinago stenura*, and comparatively analysed 20 mitogenomes of Charadriiformes. The mitogenomes display moderate size variation, and most of variation due to mutations in the control region. In 13 protein-coding genes, we found: 1. The GC skews are always negative, while the negative AT skews are found in 5 genes, 2. The average uncorrected pairwise distances reveal heterogeneity of evolutionary rate for each gene, 3. The ATG and TAA, respectively, are observed the most commonly start and stop codon. The highest dN/dS is detected for ATP8 (0.16) among Charadriiformes, while the lowest for COI (0.01), indicating that 13 protein-coding genes are evolving under the purifying selection. Predicted secondary structures of tRNAs indicate that the sequences and structures of anticodon, amino acceptor, and TψC arms are highly conserved, and most nucleotide variation is restricted to dihydrouridine arms with obvious indel polymorphisms. A total of 15 conserved sequence boxes were recognized in the control regions, and the 4 bp (5’-AAAC-3’) and 7 bp (5’- AAACAAC -3’) repeat sequences occurred frequently. Phylogenomic analysis based on the nearly complete mitochondrial genomes strongly supported the monophyly of the order, and the suborder Charadrii is at the basal of Charadriiformes. Moreover, our results well resolved the complexity family-level relationships and clearly depicted the evolutionary processes of Charadriiformes, based on 12 mitochondrial protein-coding genes from 18 families. This study improves our understanding of mitogenomic structure and evolution, which can provide further insights into our understanding of phylogeny and taxonomy in Charadriiformes.

## Introduction

**Fig 1:**
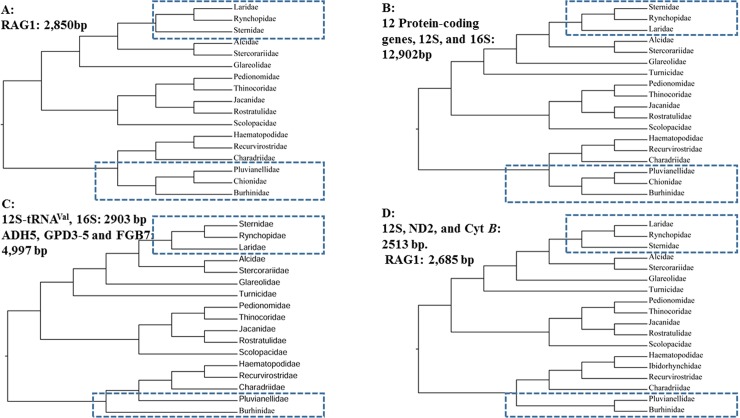
Family-level phylogenetic relationships of Charadriiformes. (A) based on RAG-1 [12], (B) based on 14 mitochondrial genes [8], (C) based on 3 mitochondrial genes and 3 nuclear genes [11], and (D) based on 3 mitochondrial genes and 1 nuclear gene [13].

The order Charadriiformes (Vertebrate: Aves), known as shorebirds, is a diverse order containing approximately 384 species belonging to 94 genera of 19 families, and has members distributing in all parts of the world. Because of the diversity in their ecology, morphology, behavior, distribution and other adaptive characters, Charadriiformes provide potential insight into evolutionary forces acting on life-history traits [1–4]. Because of convergent evolution, the use of morphological and biochemical methods to reconstruct Charadriiformes phylogeny is unresolved and poorly supported [5, 6]. Based on the genetic analysis (mitochondrial and/or nuclear genes), the majority of previous analyses revealed that the order can be divided taxonomically into three major clades (Charadrii, Scolopaci, and Lari), and place the Charadrii as sister to the other two groups [7–11]. However, the complexity family-level relationships of Charadriiformes have not been well resolved, especially in the deep nodes of the phylogenetic trees (Fig 1). For example, in the major clade of Lari, many studies suggest that the Sternidae and Rynchopidae are nearest phylogenetic neighbors (Fig 1B and 1C), others place the Sternidae as sister to the other two families (Fig 1A and 1D). Another potential conflict in topology is the major clade of Charadrii, the relationship of Chionidae, Pluvianellidae and Burhinidae (Fig 1).

Animal mitogenome is an approximately 16 kb circular molecules [14]. This genome typically contains 13 protein-coding genes, 2 ribosomal RNAs (12S rRNA and 16S rRNA), 22 transfer RNAs (tRNAs), and a non-coding control region (CR) [15, 16]. The genome-level characteristics of mitogenomes include the base composition, codon usage, gene order arrangement, tRNA and rRNA gene secondary structures, and the signaling genes control of replication and transcription [14, 17]. Such information has been used to show potential in resolving ancient patterns of evolutionary history, and also for developing conservation management programs for endangered species [8, 18, 19]. In the past decade, the number of sequenced mitogenomes have accumulated and these sequences have been widely used in biological research, especially when studying molecular evolution, genetic structure, and phylogeography [20–27]. Especially, a number of studies performed using mitochondrial genome sequences to elucidate avian phylogeny [21, 22, 28, 29].

Although some studies have used a few mitochondrial and/or nuclear genes in reconstructing family-level phylogeny within Charadriiformes [4, 8, 11–13, 30], mitochondrial genome sequences and structures have proven to be useful for elucidation of evolutionary relationships, and the comprehensive study of the entire mitogenomes within Charadriiformes are still not well studied. Despite recent rapid increases in the available information on bird mitogenomes, only 19 complete mitogenomes sequences have been reported in database of Charadriiformes (Table 1). Of these sequenced mitogenomes, 5 were from species in the suborder Charadrii (Haematopodidae, Recurvirostridae and Charadriidae), 5 in Scolopaci (Jacanidae and Scolopacidae), and 9 in Lari (Laridae, Stercorariidae and Alcidae).

**Table 1:** List of the complete mitogenomes sequences included in this study.

Suborder	Family	Species	Accession no.	Size (bp)
Charadrii	Haematopodidae	*Haematopus ater*	AY074886	16791
	Recurvirostridae	*Recurvirostra avosetta*	KP757766	16897
	Charadriidae	*Vanellus cinereus*	KM404175	17074
		*Vanellus cinereus*	KM873665	17135
		*Vanellus vanellus*	KM577158	16795
Scolopaci	Jacanidae	*Jacana jacana*	KJ631049	16975
		*Jacana spinosa*	KJ631048	17079
	Scolopacidae	*Scolopax rusticola*	KM434134	16984
		*Arenaria interpres*	AY074885	16725
		*Eurynorhynchus pygmeus*	KP742478	16707
		*Gallinago stenura*	KY056596 ^a^	16899
Lari	Laridae	*Chroicocephalus brunnicephalus*	JX155863	16769
		*Chroicocephalus ridibundus*	KM577662	16807
		*Chroicocephalus saundersi*	JQ071443	16725
		*Larus crassirostris*	KM507782	16746
		*Larus dominicanus*	AY293619	16701
		*Ichthyaetus relictus*	KC760146	16586
		*Sternula albifrons*	KT350612	16357
	Stercorariidae	*Stercorarius maccormicki*	KM401546	16669
	Alcidae	*Synthliboramphus antiquus*	AP009042	16730

^a^ The mitogenome sequence (KY056596) was obtained in this study.

In this study, we sequenced and annotated the complete mitogenome of the pin-tailed snipe (*Gallinago stenura*) from the family of Scolopacidae, and this species is most common migrant snipe. We compared the newly sequenced mitogenome with previously published sequences, we tried to address: (1) features of Charadriiformes mitogenomes, (2) rates and patterns of mitochondrial gene evolution within Charadriiformes, and (3) the adaptation of the Charadriiformes mitogenome to different environment based on dN/dS analysis. And then, we have collected sequences of 12 protein-coding genes from 18 families to assess the family-level relationships of Charadriiformes. This study contributes to more molecular information on mitogenomic structure and evolution, which can provide further insights into our understanding of phylogeny and taxonomy in Charadriiformes.

## Materials and methods

### Ethical statement

No specific permits were required for the specimens collected for this study. The specimen was common in China and the field studies did not involve endangered or protected species. Our experimental procedures complied with the current laws on animal welfare and research in China, and were specifically approved by the Animal Research Ethics Committee of Nanjing Normal University.

### Sample collection and DNA extraction

The muscle tissue sample of *G*. *stenura* was collected from a derelict and abandoned mist net in Rudong, Jiangsu Province, China (32°32'43.41" N, 121°06'09.01" E). The tissue was preserved in absolute ethanol and stored at −80°C in our laboratory at Nanjing Normal University (specimen voucher: NJNU-Gst01). Total genomic DNA was extracted using standard phenol-chloroform methods [31].

### Primer design, PCR amplification and sequencing

The primers were designed based on sequence-conserved regions, which were identified using multiple alignments of the complete mitogenomes of 19 closely related sequences downloaded from GenBank (Table 1). Using the primer pairs newly designed for this study, PCR amplifications were carried out in a final reaction volume of 25 μL, which contained 10 × buffer 3 μL, 25 mmol/L MgCl_2_ 2 μL, 2 mmol/L dNTPs 2 μL, 10 μM of each primer (forward and reverse) 1 μL, 1 μL template DNA, 1U Taq DNA polymerase (Takara, Japan), and sterile double-distilled water (ddH_2_O) to make up a final volume of 25 μL. Amplifications were performed in an Applied Biosystems 2720 thermal cycler with heated lid under the following conditions: 95°C for 5 min (initial denaturation); then 35 cycles of 95°C for 30 s (denaturation), 50–55°C for 30 s (annealing), and 72°C for 1 min (extension); and a final extension at 72°C for 8 min and 4°C hold. Annealing temperature was changed from 50 to 55°C to improve the quality of PCR products as necessary. PCR products were excised by electrophoresis on a 1% agarose gel and visualized by Ethidium Bromide staining. And then the PCR products were purified using a gel extraction kit (Promega) and sequenced with each of the PCR primers on an ABI 377 sequencer.

### Genome annotation and sequence analysis

Sequences obtained were assembled and edited using the software SeqMan (DNAStar, Inc.) to generate complete mitochondrial DNA sequences. The protein-coding genes were determined by open reading frame finder implemented at the NCBI website with the vertebrate mitochondrial genetic code, and then finally confirmed by sequence comparisons with the reported Charadriiformes mitogenomes. The tRNAscan-SE 1.21 [32], MITOS [33], and ARWEN [34] were used to confirm tRNA annotation results. The skewing of the nucleotide composition was calculated according to the following formulas: AT skew = (A–T) / (A + T) and GC skew = (G − C) / (G + C) [35, 36]. The tandem repeats were searched in the CR using the Tandem Repeats Finder program [37].

The number of variable sites, the parsimony informative sites, the singleton, and the average uncorrected pairwise distances for each protein-coding gene were calculated by MEGA 6.0 [38]. The rates of non-synonymous substitutions (Ka, *π* modified), and synonymous substitutions (Ks, *π* modified) for each protein-coding genes were determined with DnaSP 5.0 [39]. The ratio of nonsynonymous substitution rate to synonymous substitution rate (ω = dN/dS) was calculated with Datamonkey [40], by choosing the vertebrate mitochondrial DNA genetic code, with in the SLAC method (*P* < 0.1). The ratio of nonsynonymous substitution rate (dN) to synonymous substitution rate (dS) is widely used as an indicator of selective pressure at the sequence level among different species. It is commonly accepted that ω > 1, ω = 1, and ω < 1 generally indicate positive selection (replacement substitutions increase fitness), neutral mutation, and negative selection (remove such substitutions from the gene pool), respectively [41].

### Phylogenetic analysis

Two datasets were assembled for phylogenetic analyses: (1) the first dataset (Dataset 1) for mitogenomic phylogeny of Charadriiformes, 13 protein-coding genes, 12S and 16S, of 20 Charadriiformes species were used (Table 1), (2) the second dataset (Dataset 2) for the family-level phylogeny of Charadriiformes, 12 protein-coding genes (with excluding ND6) of 40 species were used (S1 Table). The two aligned datasets were 13,965 and 10,773 bp in length for the Dataset 1 and Dataset 2 matrices, respectively. In all phylogenetic analyses, *Columba livia*, *Gallus gallusa*, *Phodilus badius*, and *Zenaida auriculata* were used as outgroups [10, 12, 42, 43]. Before reconstructing the phylogenetic trees, sequence alignment was carried out. Each mitochondrial gene was aligned individually using Muscle in MEGA 6.0 [38]. The 13 protein-coding genes were translated into amino acids, and then aligned using Muscle in MEGA 6.0 [38] with default parameters for each gene, and finally retranslated into nucleotide sequences after removing the stop codons.

The phylogenetic trees were inferred under the maximum likelihood (ML) and Bayesian inference (BI), which were performed with RAxML 8.2.4 [44] and MrBayes 3.2.2 [45], respectively. To determine the optimal partitioning of the data, we explored three partition schemes in phylogenetic analyses. The data set was partitioned *a priori* on the basis of genes and codon positions. The three partitioning schemes are: (1) single (not partitioning), (2) each gene as one partition, (3) partitioned by each of the three codon positions in each gene. The best-fit partitioning scheme and the most appropriate nucleotide evolution model for each partition were implemented in PartitionFinder 1.1.1 [46]. Unpartitioned scheme using a GTR+ *I* + *G* substitution model. For ML analysis, node support was calculated with a GTRGAMMA model via rapid bootstrapping (-f a -x option) with ten runs and 1,000 replications to estimate the best topology. For the BI analysis, two independent runs of four Markov Chains Monte Carlo (MCMC) chains (one cold chain and three hot chains) were simultaneously run for 2.0 × 10^7^ generations, with sampling conducted every 1000 generations. The first 25% of the initial trees were discarded as ‘‘burn-in” and the remaining trees were used to calculate 50% majority rule consensus tree and Bayesian posterior probabilities. The MCMC runs were repeated twice to confirm consistent approximation of the posterior parameter distributions. The convergence of MCMC runs and effective sample sizes (ESS > 200) were checked by plotting the log likelihood scores against the generation times using the program Tracer 1.6 (http://beast.bio.ed.ac.uk/Tracer).

## Results and discussion

### Genome organization

**Fig 2:**
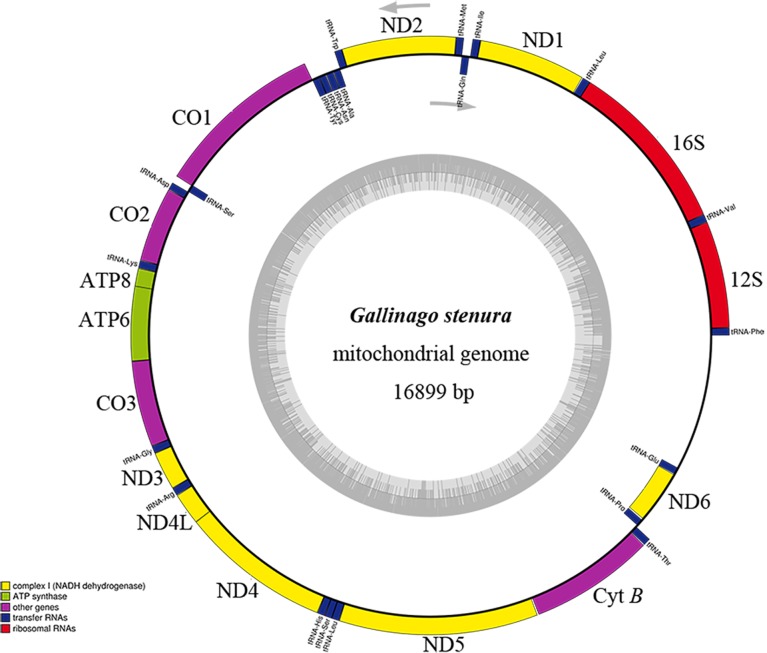
Circular map of the mitochondrial genome of *Gallinago stenura*.

The mitogenome of *G*. *stenura* is a closed circular molecule of 16,899 bp in length (GenBank No. KY056596), and its map is shown in Fig 2. The completely sequenced mitogenomes from Charadriiformes encode a CR, and a typical set of 37 mitochondrial genes containing 13 protein-coding genes, 2 rRNA genes (12S rRNA and 16S rRNA), and 22 tRNA genes. Gene arrangement is highly conserved within the Charadriiformes mitogenomes, without showing any structural rearrangement [10, 14]. The gene of ND6 and 8 tRNA are encoded on the light strand, whereas the other genes are located on the heavy strand (S2 Table). The mitogenomes of the twenty completely sequenced Charadriiformes species displayed moderate size variation, the mean size was 16,807 bp (SD = 179.66, n = 20), ranging from 16,357 bp (*Sternula albifrons*) to 17,135 bp (*Vanellus cinereus*) (Table 1). Most of the size variation primarily due to length mutation in the CR. In the *G*. *stenura* mitogenome, gene overlaps occurred 10 times, spanning 1–10 nucleotides, for a total of 35 nucleotides. The longest overlap (10 bp) existed between ATP8 and ATP6, and the pairs of COI/tRNA^Ser^ overlap 9 nucleotides, whereas 5 pairs (16srRNA/tRNA^Leu^, tRNA^Gln^/tRNA^Met^, tRNA^Cys^/tRNA^Tyr^, ATP6/COIII and tRNA^Ser^/tRNA^Leu^) overlap only one nucleotide (S2 Table). The intergenic spacer regions occurred 17 times, spanning 1–12 bp, for a total of 69 bp (S2 Table).

### Nucleotide composition

**Fig 3:**
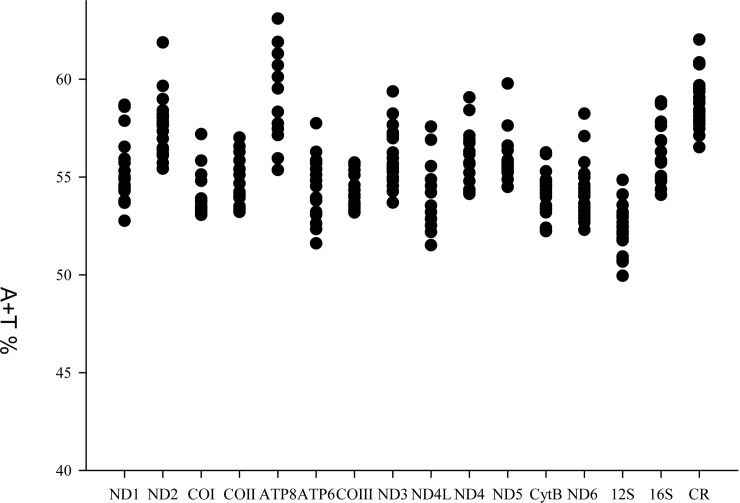
The A + T content in Charadriiformes mitogenomes. Each point represents a species.

The overall mean base composition of Charadriiformes mitogenomes was: A, 31.20%; C, 30.60%; T, 24.44% and G, 13.76%. Twenty Charadriiformes mitogenomes are consistently biased towards AT rich, ranging from 54.39% (*Chroicocephalus saundersi*) to 58.35% (*G*. *stenura*) (Table 2), which is consistent with previous avian mitogenomes [22]. Interestingly, higher A + T content is observed not only in the CR but also in protein-coding genes and rRNA (Fig 3). The lowest A + T content is found in the gene of 12S rRNA (19 species) or ND1 (only in *Scolopax rusticola*), whereas the highest A + T content is found in ATP8 (11 species) or CR (9 species).

**Table 2:** Nucleotide composition of mitochondrial genomes in Charadriiformes.

		Proportion of nucleotides (%)						
Species	Accession no.	A	T	G	C	AT content	AT skew	GC skew
*Haematopus ater*	AY074886	31.59	23.62	13.67	31.12	55.21	0.14	-0.39
*Recurvirostra avosetta*	KP757766	31.72	23.59	13.56	31.13	55.31	0.15	-0.39
*Vanellus cinereus*	KM404175	31.63	23.53	13.77	31.08	55.15	0.15	-0.39
*Vanellus cinereus*	KM873665	31.46	23.69	13.98	30.87	55.14	0.14	-0.38
*Vanellus vanellus*	KM577158	31.44	24.03	13.76	30.77	55.47	0.13	-0.38
*Jacana jacana*	KJ631049	31.88	24.35	13.15	30.62	56.23	0.13	-0.40
*Jacana spinosa*	KJ631048	31.54	24.86	13.09	30.42	56.45	0.12	-0.40
*Scolopax rusticola*	KM434134	31.79	25.02	13.34	29.85	56.81	0.12	-0.38
*Arenaria interpres*	AY074885	30.64	24.71	13.94	30.72	55.34	0.11	-0.38
*Eurynorhynchus pygmeus*	KP742478	31.29	24.85	13.84	30.02	56.14	0.11	-0.37
*Gallinago stenura*	KY056596	32.21	26.14	12.88	28.77	58.35	0.10	-0.38
*Chroicocephalus brunnicephalus*	JX155863	30.70	24.03	14.16	31.11	54.73	0.12	-0.37
*Chroicocephalus ridibundus*	KM577662	30.81	23.97	14.16	31.06	54.78	0.12	-0.37
*Chroicocephalus saundersi*	JQ071443	30.41	23.98	14.39	31.21	54.39	0.12	-0.37
*Larus crassirostris*	KM507782	30.62	24.34	14.13	30.91	54.96	0.11	-0.37
*Larus dominicanus*	AY293619	30.54	24.45	14.13	30.88	54.98	0.11	-0.37
*Ichthyaetus relictus*	KC760146	30.62	24.38	14.07	30.93	55.00	0.11	-0.37
*Sternula albifrons*	KT350612	31.11	26.12	13.74	29.03	57.22	0.09	-0.36
*Stercorarius maccormicki*	KM401546	30.94	24.39	13.82	30.85	55.34	0.12	-0.38
*Synthliboramphus antiquus*	AP009042	31.10	24.75	13.56	30.60	55.85	0.11	-0.39

**Fig 4:**
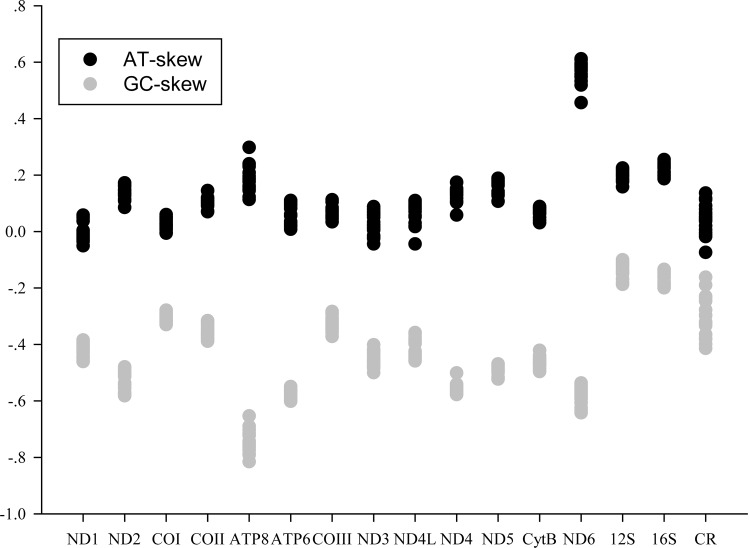
The AT skew and GC skew in Charadriiformes mitogenomes. Each point represents a species.

AT and GC skews are a measure of compositional asymmetry. In Charadriiformes mitogenomes, AT skews values were always positive, while the values of GC skew were negative. The AT skew value observed is 0.12 ± 0.02 (mean ± SD), ranging from 0.09 (*Sternula albifrons*) to 0.15 (*Recurvirostra avosetta*). The GC skews value is −0.38 ± 0.01, ranging from −0.40 (*Jacana spinose*) to −0.36 (*S*. *albifrons*) (Table 2). The lowest value of GC skew was always found in Jacanidae (−0.40), and highest value was found in Laridae (−0.36 to −0.37). In general, AT and GC skews in Charadriiformes mitogenomes are similar to patterns typically found in most animal mitogenomes, which positive AT skew and negative GC skew are found for H-strand, implying the specific bias toward A and C in nucleotide composition [47, 48]. The GC skew in Charadriiformes mitogenomes revealed that the ATP8 containing significantly higher skews than other regions, and the positive GC skew was not found in this work. However, there was a marked negative AT skew in ND1 (11 species), COI (three species), ND3 (five species), ND4L (one species) and CR (four species) on the H-strand (Fig 4).

### Protein-coding genes evolutionary rate

Total length of the protein-coding genes in each species was 11,399 bp after removing termination codons and indels. Indels were only found in ATP8, COIII, ND5, and Cyt *b*, and ranged from two to five amino acids. Comparison of each protein-coding gene provides a better understanding of the evolutionary patterns of molecular evolution. The length of the 13 protein-coding genes of Charadriiformes genomes showed that the gene of ND5 and ATP8 being the longest and shortest, respectively. The percent of variable positions in each gene ranged from 33.91% (COI) to 53.57% (ATP8), and the parsimony informative sites ranged from 28.32% (COIII) to 51.6% (ATP8). The analysis of variable sites showed that the ATP8 contained more variable sites. The average uncorrected pairwise distances revealed the heterogeneity of evolutionary rate for each gene, the COIII (0.13), COI (0.14) and COII (0.14) had slow evolutionary rate, whereas those of ND6 (0.20), ND2 (0.20) and ATP8 (0.24) relatively faster (Table 3). So, we can infer that the COIII was the most conserved protein-coding gene, and ATP8 the least conserved. At the nucleotide levels, three genes (COI, COII and COIII) had the lowest evolutionary rates, suggesting that they are useful candidate barcoding markers. The nucleotide composition had a positive correlation with the observed variation in each gene, indicating that the larger biased in nucleotide composition might be expected evolving more quickly (Fig 4, Table 3).

**Table 3:** The mutational information and average distances among Charadriiformes calculated by gene.

Gene	Length (bp)	%Vs	%Pis	%S	%Aupd
ND1	978	55.01	44.99	37.42	19.00
ND2	1041	51.30	48.70	38.33	20.30
COI	1551	66.09	33.91	28.69	14.40
COII	684	64.04	35.96	30.12	14.40
ATP8	168	46.43	53.57	43.45	23.50
ATP6	684	55.12	44.88	36.11	18.20
COIII	784	65.43	34.57	28.32	13.30
ND3	351	58.40	41.88	35.33	18.00
ND4L	297	53.20	46.80	37.37	17.90
ND4	1378	54.86	45.14	35.70	17.90
ND5	1818	51.76	48.07	37.24	18.80
Cyt *b*	1143	60.89	39.11	30.53	15.10
ND6	522	50.19	49.81	36.40	19.80

Vs: variable sites; Pis: parsimony informative sites; S: singleton; Aupd: The average uncorrected pairwise distances.

**Fig 5:**
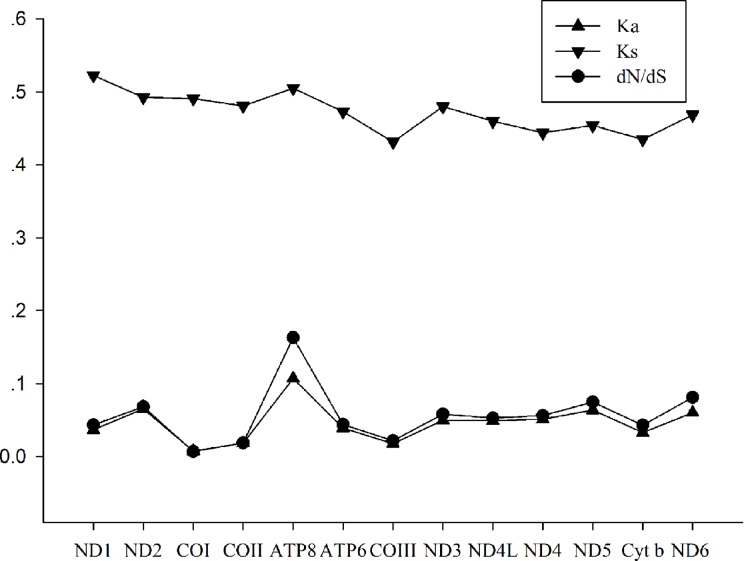
Evolutionary rates of 13 protein-coding genes in Charadriiformes mitogenomes. The Y-axis provides the substitution rate of mitochondrial gene. Synonymous nucleotide substitutions per synonymous site (Ks) and nonsynonymous nucleotide substitutions per nonsynonymous site (Ka) are calculated using Dnasp, and dN/dS is calculated using DataMonkey.

To better understand the evolutionary patterns among the 13 protein-coding genes and the role of selection in Charadriiformes species, the values of Ka, Ks, and dN/dS (ω) were calculated for each protein-coding gene, respectively (Fig 5). The Ks of ND1 was the highest, while the values of Ka and ω for ATP8 were the highest. The highest dN/dS was detected for the ATP8 gene (0.16) among Charadriiformes, while the lowest for the COI gene (0.01). The dN/dS values for all protein-coding genes were far lower than one (≤ 0.16), indicating that these genes were evolving under the purifying selection.

### The usage of start and stop codon

**Fig 6:**
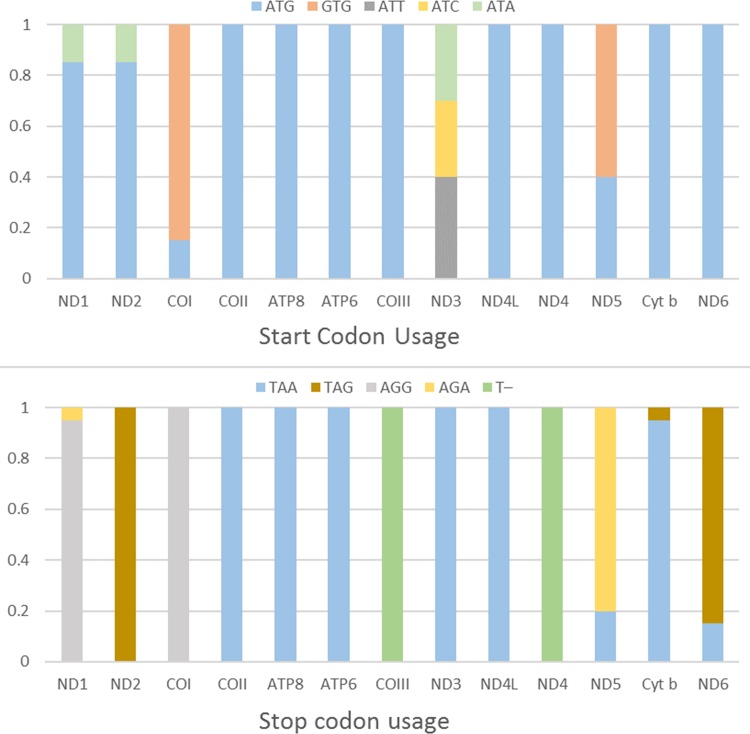
The usage of start and stop codons in the 13 mitochondrial protein-coding genes of the order Charadriiformes. All genes are shown in the order of occurrence in the mitochondrial genome starting from ND1.

Start and stop codons vary among the protein-coding genes in Charadriiformes, although ATG and TAA were the most commonly observed. Start and stop codon usage biases were shown in Fig 6. Five start codons (ATG, GTG, ATT, ATC, and ATA) were detected in the 13 protein-coding genes. The most common start codon was ATG, which accounts for 78.85% of the start codons, followed by GTG (11.15%). The start codon ATG appeared in 12 protein-coding genes (with the exception of ND3), and 8 genes (COII, ATP8, ATP6, COIII, ND4L, ND4, Cyt *b*, and ND6) only used ATG as start codon. The start codon GTG was commonly used in the COI and ND5. Then the start codons ATT and ATC were only used in the ND3. The start codon ATA were found in the ND1, ND2, and ND3 (Fig 6), which was frequently observed in other avian orders [20]. While in the suborder Lari, the start codons ATC and ATA were not common used, as they were only found in the ND3 of *S*. *antiquus* and *A*. *interpres* [10].

Four stop codons (TAA, TAG, AGG, and AGA) and an incomplete stop codon (T–) were detected. The most common stop codon TAA served as stop codon only in six genes (COII, ATP8, ATP6, ND3, ND4L, and Cyt *b*) (Fig 6). In the case of other bird orders, the stop codon TAA appears only in COII, ND4L, and Cyt *b* [20]. The stop codon TAG appeared in the ND2 and ND6. But the stop codon TAG has so far been described in the Cyt *b* of *Aceros corrugatus* (Coraciiformes; Bucerotidae) and the ND6 of a few Columbidae species [20]. The stop codon AGG was found in COI and ND1 (Fig 6). The stop codon AGA was found in ND5, and ND1. The stop codon T–only appeared in the COIII and ND4. Truncated stop codons are common and may be completed by a poly-adenylation of the 3’-end of the mRNA *via* post-transcriptional [49–51].

In the gene of ND3, an extra nucleotide (C: cytosine) was present at the position 174 of 16 species, which was not observed in four species (*Chroicocephalus ridibundus*, *C*. *saundersi*, *Synthliboramphus antiquus* and *Scolopax rusticola*). In the species of *C*. *saundersi*, Yoon et al. (2015) found the extra base in ND3, whereas Ryu and Kwang (2012) lacked it [10, 52]. The extra base insert in ND3 has been observed in some birds and turtles [53], but it appears to be randomly happened in closely related species, even within species [10, 52]. So, we can infer that there is no evolutionary significance for the extra insertion site.

### Sequence features of rRNA and tRNA genes

As other avian rRNA genes, Charadriiformes mitogenomes had 2 rRNA (12S and 16S) that were identified on the heavy strand and located at a conserved position between tRNA^Phe^ and tRNA^Leu^ genes, and separated by tRNA^Val^ gene (Fig 2; S2 Table). The length of 12S rRNA varied from 963 bp (*Haematopus ater*) to 981 bp (*Vanellus cinereus*) (KM404175), and the smallest and largest 16S rRNA genes were 1,589 bp (*Jacana spinose* and *J*. *jacana*) and 1,607 bp (*V*. *vanellus*). The average size of two combined rRNA genes was 2,566 bp (±8, n = 20), and ranged from 2,553 bp (*H*. *ater*) to 2,580 bp (*V*. *vanellus*). Therefore, there was no substantial size variation between rRNAs, since their sequence size varied slightly with few base pairs difference. In *G*. *stenura*, the combined size of the two rRNA genes was 2,571 bp.

Twenty-two tRNA genes were identified in Charadriiformes in the same genomic positions as observed in Laridae [10], with an average size ranging from 65.0 ± 0.9 bp in tRNA^Ser (AGY)^ to 74.0 ± 0.2 bp in tRNA^Leu (UUR)^. The length difference was due to the mutation of stem and loop sizes of the dihydrouridine (DHU) and TΨC arms. In *G*. *stenura* mitogenome, the length of 22 tRNA genes ranged from 66 bp [tRNA^Ser(AGY)^] to 74 bp [tRNA^Ser^ and tRNA^Glu^] (S2 Table). Putative secondary structures of tRNAs in *G*. *stenura* indicated that 21/22 [except for tRNA^Ser (AGY)^, which appears to lack the DHU arm] possess a typical canonical cloverleaf secondary structure, which were similar to those of other Charadriiformes species, indicated their similar functions (S1 Fig). The sequences and structures of anticodon, amino acceptor, and TψC arms were highly conserved, while the nucleotide variation was mostly restricted to DHU arms with obvious indels polymorphisms (13/22 tRNA genes; S2 Fig).

### Control region

The CR of Charadriiformes mitogenomes were highly divergent, such as the length ranged from 800 bp in *Sternula albifrons* to 1,563 bp in *V*. *cinereus*. However, the CR duplication was not found in this study. In CR of *G*. *stenura*, the nucleotide composition was 31.97% A, 28.78% T, 11.80% G, and 27.45% C, and with an A + T bias of 60.76%. The AT skew of CR ranged from −0.07 in *Ichthyaetus relictus* to 0.12 in *V*. *cinereus*, and there were two species (−0.07 in *I*. *relictus* and −0.02 in *Larus dominicanus*) with a negative value. The mean value of GC skew was −0.31, ranging from −0.41 in *S*. *rusticola* to −0.16 in *V*. *cinereus* (KM873665).

**Fig 7:**
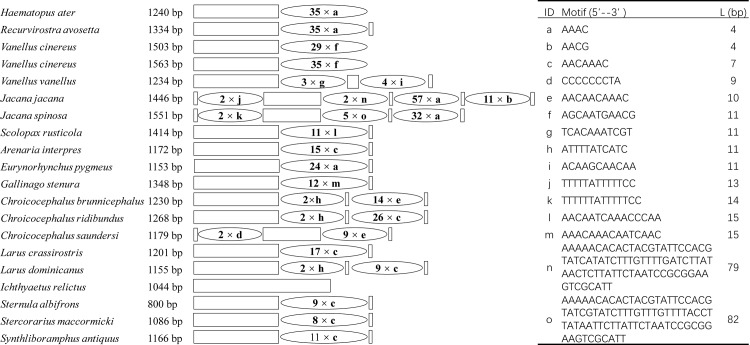
Organization of the control region in Charadriiformes mitochondrial genomes. The location and copy number of tandem repeats are shown by oval with Arabic numerals inside. Non-repeat regions are indicated by square box. L: Length.

We observed large tandem repeats with two or more copies in CR with the exception of *I*. *relictus*. In most species (12/20), tandem repeat sequences only had one type of repeat unit. Tandem repeat sequences contained two types of repeat units in 5 species (*V*. *vanellus*; *C*. *brunnicephalus*, *C*. *ridibundus*, *C*. *saundersi*, and *L*. *dominicanus*). The sequences contained three and four types in *Jacana spinosa* and *J*. *jacana* respectively (Fig 7). Total of 15 kinds of tandem repeats were found in CR. The motifs of 5’-AAACAAC-3’ (occurs in 7 species) and 5’-AAAC-3’ (in 5 species) are most common. However, 10/15 motifs were only found in one species, suggesting the fast rate evolution in the CR. Two long motifs of n (79 nucleotides with 2 tandem repeats) and o (82 nucleotides with 5 tandem repeats) only occurred in *Jacava* (Fig 7). Interestingly, the five motifs (a, c, f, l and m) had large copies (Fig 7), which had a strictly conserved common sequence (5’-AAAC-3’). The CR of Charadriiformes mitogenome shows distinct sequence and structural characteristics, such as varying sizes and diverse tandem repeats, which are taxon-specific and can potentially be used as genetic markers for evolution and population genetic studies.

### Phylogenetic analysis

**Fig 8:**
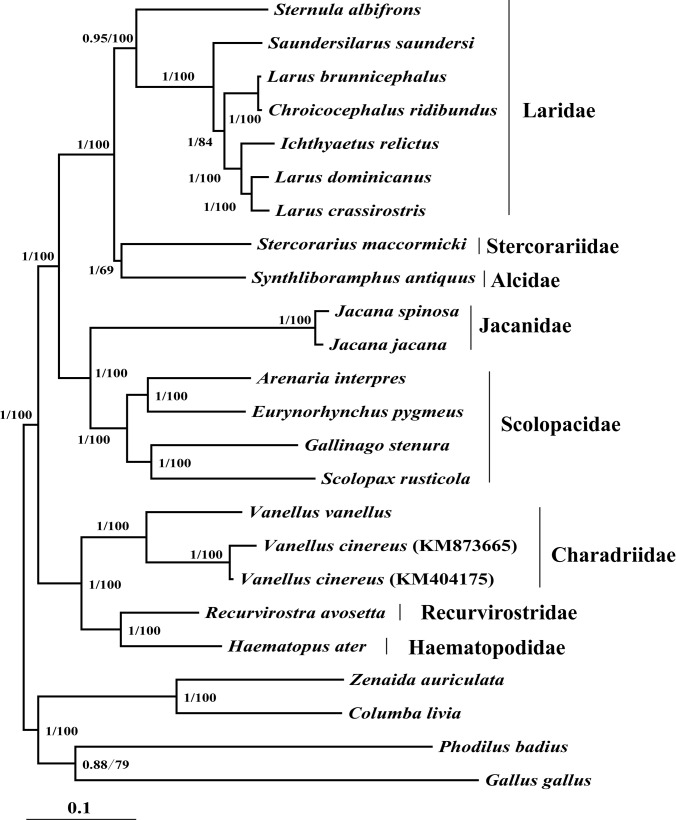
The phylogenetic trees of Charadriiformes based on the 13 mitochondrial protein-coding genes, 12S and 16S dataset using BI and ML methods. Branch lengths and topologies were obtained using Bayesian inference analysis, and the numbers above each branches are the posterior probabilities and ML bootstrap support.

Phylogenetic analysis with two inference methods (ML and BI) of 13 mitochondrial protein-coding genes, 12S and 16S (Dataset 1) for 20 Charadriiformes species revealed the identical topologies, which were highly supported by bootstrap and posterior probabilities at most nodes (Fig 8). Relationships of the order Charadriiformes based on the nearly complete mtDNA strongly support monophyly of the order Charadriiformes.

**Fig 9:**
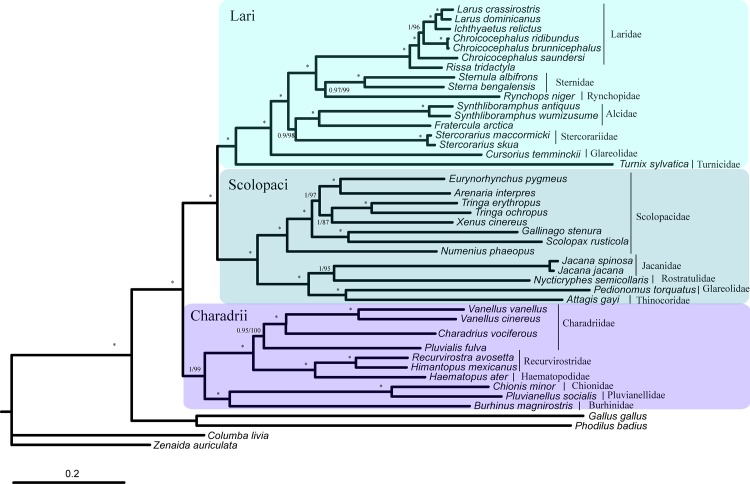
The phylogenetic trees of Charadriiformes based on the concatenated 12 mitochondrial protein-coding genes using BI and ML methods. Branch lengths and topologies were obtained using Bayesian inference analysis. Asterisks indicate bootstrap values of 100% and posterior probability of 1.

The topologies recovered using ML and BI of the combined 12 protein-coding genes (Dataset 2, 10,773 bp after alignment) were consistent (Fig 9). These relationships were well supported, as the posterior probabilities in the Bayesian tree are 1 at all nodes in the tree excepting two nodes, and similarly of the maximum likelihood bootstrap values. The order was divided into three major clades including Lari (Laridae, Sternidae, Rynchopidae, Alcidae, Stercorariidae, Glareolidae, and Turnicidae) and its sister Scolopaci (Scolopacidae, Jacanidae, Rostratulidae, Pedionomidae, and Thinocoridae), which were in turn sister to the suborder Charadrii (Charadriidae, Recurvirostridae, Haematopodidae, Chionidae, Pluvianellidae, and Burhinidae) (Fig 9), as previous analysis based on both nuclear and mitochondrial gene have indicated [8, 11–13, 30]. Within Lari, our phylogenies indicated that Laridae was paraphyletic to Sternidae plus Rynchopidae. This result is in congruence with previous comprehensive phylogenetic analyses in Charadriiformes, using a combination of mitochondrial and nuclear genes [8, 11]. The Alcidae plus Stercorariidae comprise the sister group to Laridae, Sternidae and Rynchopidae. And Glareolidae and Turnicidae are successive sister groups to these clades instead as basal members of the major clade Lari. Within Scolopaci, Rostratulidae and Jacanidae as a sister group to the Pedionomidae plus Thinocoridae, and Scolopacidae is the basal to the other. This result is in congruence with more and more recent studies [11, 13, 54]. Within Charadrii, Burhinidae is sister to Chionidae plus Pluvianellidae, Charadriidae is paraphyletic to Haematopodidae plus Recurvirostridae (Fig 9). According to the phylogenetic results, the monophyly of Charadriiformes was strongly supported in ML and BI analyses.

## Conclusion

In this study, we sequenced and annotated the complete mitogenome of *Gallinago stenura*. We compared 20 Charadriiformes mitogenomes to illustrate mitogenomes structure and investigate their evolutionary relationship. The Charadriiformes mitogenomes display moderate size variation, the mean size was 16,807 bp (SD = 179.66, n = 20), and most of the size variation due to mutations in the CR. Nucleotide composition is consistently biased towards AT rich, and the A + T content also varies for each protein-coding genes, such as the variation in ATP8 is the highest, whereas the variation in COIII is the lowest. In the 16 gene regions, the GC skew is always negative, with the ATP8 containing significantly higher value than other regions. However, the marked negative AT skew is found in 5 gene regions (ND1, COI, ND3, ND4L and CR). The average uncorrected pairwise distances reveal heterogeneity of evolutionary rate for each gene, the COIII, COI and COII have slow evolutionary rate, while it is relative fast for the ND6, ND2 and ATP8. In 13 protein-coding genes, Charadriiformes mitogenomes contain 5 kinds of start codons (ATG, GTG, ATT, ATC, and ATA), four stop codons (TAA, TAG, AGG, and AGA), and an incomplete stop codon (T–). The codon usage analysis revealed that the ATG and TAA are the most common start and stop codon, respectively. The start codon ATG appears in 12 protein-coding genes (with the exception of ND3) and 8 genes only used ATG as start codon. The stop codon TAA appears in 8 genes, and 6 genes (COII, ATP8, ATP6, ND3, ND4L, and Cyt *b*) exclusively used TAA as stop codon. Putative secondary structures of tRNAs indicate that the sequences and structures of anticodon, amino acceptor, and TψC arms are highly conserved, and most of the nucleotide variation is restricted to DHU arms with obvious indel polymorphisms. In the CR, there are 15 kinds of tandem repeats, 4 bp (5’-AAAC-3’) and 7 bp (5’- AAACAAC -3’) repeat sequences occur frequently. Phylogenomic analysis based on the nearly complete mitochondrial genomes strongly supports the monophyly of the order Charadriiformes, with the suborder Lari sister to the Scolopaci, which is in turn a sister group to the suborder Charadrii. Moreover, our results well resolved the complexity family-level relationships and clearly depicted the evolutionary processes of Charadriiformes, based on 12 mitochondrial protein-coding genes from 18 families. In future, sequencing more mitogenomes from various taxonomic levels will significantly improve our understanding of mitogenomic evolution and phylogenetic relationships in Charadriiformes.

## Supporting information

S1 Table Taxon sampling and GenBank accession numbers. (XLSX)

S2 Table Gene organization of *Gallinago stenura* mitochondrial genome. (DOCX)

S1 Fig Predicted secondary structures of the 22 tRNA genes identified in the mitogenome of *G*. *stenura*. All tRNA genes are shown in the order of occurrence in the mitochondrial genome starting from tRNA^Phe^.
(TIF)

S2 Fig The alignment and predicted secondary structures of the 22 tRNA genes identified in the Charadriiformes mitogenomes. (TIF)
