# Supplementary material for: The mitochondrial genome of pin-tailed snipe Gallinago stenura, and its implications for the phylogeny of Charadriiformes
Source: PLoS One. 2017 Apr 6;12(4):e0175244. doi: 10.1371/journal.pone.0175244 (PMC5383286; doi:10.1371/journal.pone.0175244)
Supplement: S2 Table — (DOCX) [file pone.0175244.s002.docx]

**S2 Table.** Gene organization of *Gallinago stenura* mitochondrial genome.

| Gene | Coding | Start | Stop | Intergentic | Overlapping | Size | No.of | AntiCodon | Start | Stop |
| --- | --- | --- | --- | --- | --- | --- | --- | --- | --- | --- |
| name | strand | position | position | nucleotides | nucleotides | (bp) | codons |  | codon | codon |
| tRNA^Phe^ | H | 1 | 68 |  |  | 68 |  | GAA |  |  |
| 12S rRNA | H | 69 | 1042 |  |  | 974 |  |  |  |  |
| tRNA^Val^ | H | 1043 | 1112 |  |  | 70 |  | TAC |  |  |
| 16S rRNA | H | 1113 | 2708 |  | 1 | 1597 |  |  |  |  |
| tRNA^Leu (UUR)^ | H | 2709 | 2782 | 12 |  | 74 |  | TAA |  |  |
| ND1 | H | 2795 | 3772 |  | 2 | 978 | 325 |  |  | AGG |
| tRNA^Ile^ | H | 3771 | 3843 | 8 |  | 73 |  | GAT | ATG |  |
| tRNA^Gln^ | L | 3852 | 3921 |  | 1 | 71 |  | TTG |  |  |
| tRNA^Met^ | H | 3922 | 3990 |  |  | 69 |  | CAT |  |  |
| ND2 | H | 3991 | 5034 |  | 2 | 1044 | 347 |  | ATA | TAG |
| tRNA^Trp^ | H | 5033 | 5102 | 1 |  | 70 |  | TCA |  |  |
| tRNA^Ala^ | L | 5104 | 5172 | 3 |  | 69 |  | TGC |  |  |
| tRNA^Asn^ | L | 5176 | 5248 | 2 |  | 73 |  | GTT |  |  |
| tRNA^Cys^ | L | 5251 | 5317 |  | 1 | 67 |  | GCA |  |  |
| tRNA^Tyr^ | L | 5317 | 5387 | 1 |  | 71 |  | GTA |  |  |
| COI | H | 5389 | 6939 |  | 9 | 1551 |  |  | GTG | AGG |
| tRNA^Ser (UCN)^ | L | 6931 | 7004 | 2 |  | 74 |  | TGA |  |  |
| tRNA^Asp^ | H | 7007 | 7075 | 1 |  | 69 |  | GTC |  |  |
| COII | H | 7077 | 7760 | 1 |  | 684 | 227 |  | ATG | TAA |
| tRNA^Lys^ | H | 7762 | 7832 | 1 |  | 70 |  | TTT |  |  |
| ATP8 | H | 7833 | 8000 |  | 10 | 168 | 55 |  | ATG | TAA |
| ATP6 | H | 7991 | 8674 |  | 1 | 684 | 227 |  | ATG | TAA |
| COIII | H | 8674 | 9457 |  |  | 784 | 261 |  | ATG | T-- |
| tRNA^Gly^ | H | 9458 | 9526 |  |  | 69 |  | TCC |  |  |
| ND3 | H | 9527 | 9878 | 4 |  | 352 | 116 |  | ATA | TAA |
| tRNA^Arg^ | H | 9883 | 9952 | 1 |  | 70 |  | TCG |  |  |
| ND4L | H | 9954 | 10250 |  | 7 | 297 | 98 |  | ATG | TAA |
| ND4 | H | 10244 | 11621 |  |  | 1378 | 459 |  | ATG | T-- |
| tRNA^His^ | H | 11622 | 11690 |  |  | 69 |  | GTG |  |  |
| tRNA^Ser (AGY)^ | H | 11691 | 11756 |  | 1 | 66 |  |  |  |  |
| tRNA^Leu (CUN)^ | H | 11756 | 11826 |  |  | 71 |  | TAG |  |  |
| ND5 | H | 11827 | 13641 | 7 |  | 1815 | 604 |  | GTG | AGA |
| Cyt *b* | H | 13649 | 14791 | 2 |  | 1143 | 380 |  | ATG | TAA |
| tRNA^Thr^ | H | 14794 | 14862 | 11 |  | 69 |  | TGT |  |  |
| tRNA^Pro^ | L | 14874 | 14943 | 9 |  | 70 |  | TGG |  |  |
| ND6 | L | 14953 | 15474 | 3 |  | 522 | 173 |  | ATG | TAG |
| tRNA^Glu^ | L | 15478 | 15551 |  |  | 74 |  | TTC |  |  |
| CR | H | 15552 | 16899 |  |  | 1348 |  |  |  |  |
