# Supplementary figures and images for: The mitochondrial genome of pin-tailed snipe Gallinago stenura, and its implications for the phylogeny of Charadriiformes
Source: PLoS One. 2017 Apr 6;12(4):e0175244. doi: 10.1371/journal.pone.0175244 (PMC5383286; doi:10.1371/journal.pone.0175244)

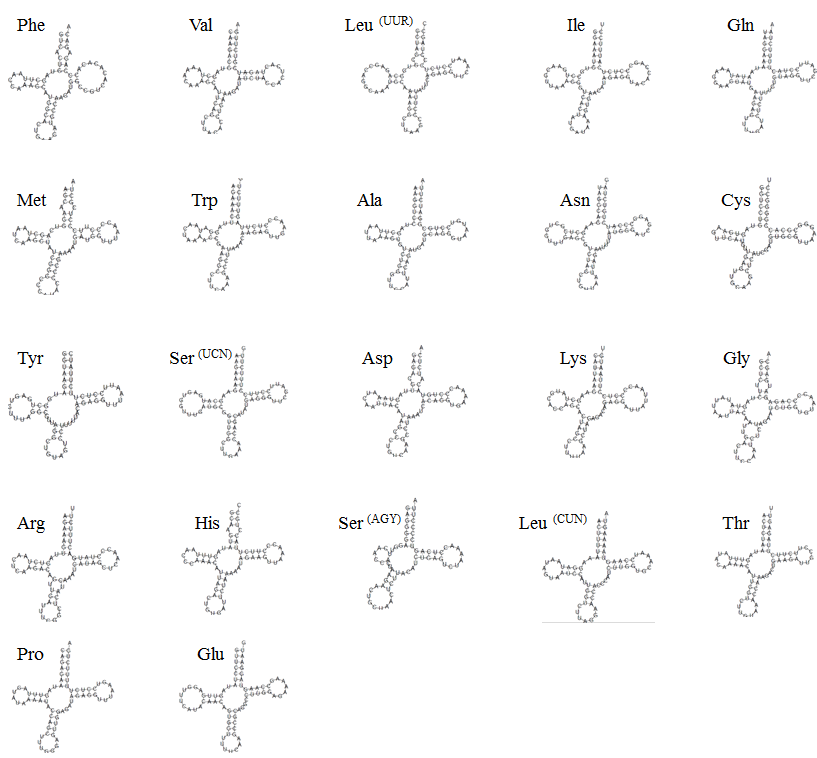

Supplement: S1 Fig — All tRNA genes are shown in the order of occurrence in the mitochondrial genome starting from tRNAPhe. (TIF) [file pone.0175244.s003.tif]
